# Multimodality Management of Skin Hyperpigmentation

**DOI:** 10.1007/s00266-025-04866-0

**Published:** 2025-04-29

**Authors:** Amer Salman Almansory, Zainab Salem Alhamdany

**Affiliations:** 1 College of Medicine, University of Basrah, Basra, Iraq; 2 Alsader Teaching Hospital, Basrah, Iraq

**Keywords:** Q-switched Nd: YAG laser, Post-inflammatory hyperpigmentation, Melasma, Hydroquinone

## Abstract

**Background:**

Skin hyperpigmentation, caused by excessive melanin production, presents both aesthetic and psychological concerns. The Q-switched Nd:YAG laser is a key treatment modality, with effectiveness influenced by factors such as skin type, laser fluence, and treatment frequency. A multimodal approach combining laser therapy and medical treatment may enhance outcomes.

**Objectives:**

This study evaluates the efficacy and safety of Q-switched Nd:YAG laser combined with medical therapy for hyperpigmentation, focusing on clinical outcomes and patient satisfaction.

**Methods:**

A randomized cross-sectional study was conducted on 300 patients in private clinics from January 2022 to September 2023. Treatment response was assessed through clinical evaluations and patient-reported satisfaction using the Likert scale. The impact of factors such as gender, sun exposure, pregnancy, and hormonal influences was analyzed.

**Results:**

The majority of participants were female (92.6%). Clinical evaluation and patient satisfaction improved significantly over successive sessions, with "Good" satisfaction ratings increasing from 80.6% initially to 98.3% at study completion. Male patients demonstrated a stronger response to treatment than females. No significant impact of the studied factors on outcomes was identified.

**Conclusions:**

A comprehensive assessment of hyperpigmentation is crucial for optimal treatment selection. A multimodal approach combining Q-switched Nd:YAG laser and medical therapy yields superior results compared to single-modality treatments, with outcomes improving progressively across sessions.

**Level of Evidence III:**

This journal requires that authors assign a level of evidence to each article. For a full description of these Evidence-Based Medicine ratings, please refer to the Table of Contents or the online Instructions to Authors  www.springer.com/00266.

## Introduction

Skin hyperpigmentation is a common dermatological condition characterized by darkened skin due to factors such as hormonal changes, inflammation, UV exposure, and medications disrupting melanocyte activity or melanin distribution [1, 2].

Although not life-threatening, it can significantly affect psychological well-being, particularly among women who often seek dermatological care after unsuccessful cosmetic treatments [3].

Melanocytes at the dermal-epidermal junction produce melanin in structures called melanosomes, and increased melanin production or melanocyte activity causes hypermelanosis [4, 5].

Conditions such as melasma, chloasma, post-inflammatory hyperpigmentation (PIH), lentigines, and freckles are categorized under hyperpigmentation [6].

Melasma and chloasma cause patchy discoloration, with the latter primarily affecting pregnant women due to hormonal changes [7–9].

PIH, more common in individuals with darker skin, often follows skin inflammation or injury such as acne, eczema, or allergic reactions [10, 11].

Lentigines ("age spots") result from sun exposure, while freckles (ephelides) are common among lighter-skinned individuals and typically appear during childhood [12, 13]. Hyperpigmentation is especially prevalent among middle-aged women and those with darker skin tones, such as African Americans, Latinos, and Middle Eastern populations, with studies indicating higher incidences among these groups [14–16].

Treatment is challenging and includes topical agents such as hydroquinone, retinoids, azelaic acid, mesotherapy, chemical peels, laser technology, and combination therapies [17].

Sunscreen with SPF 30–60 is essential for preventing recurrence and reducing UV damage [18].

Laser treatments, including intense pulsed light, Q-switched lasers, and picosecond lasers, target melanin with promising but debated outcomes, especially for darker skin tones [19–26]. Despite advancements, a definitive cure remains elusive, emphasizing the need for continued research and tailored treatment approaches [20, 21].

### Aim of the Study

The study aims to assess the effectiveness and safety of treatments for skin hyperpigmentation, using multimodality treatment, including Q-switched neodymium: yttrium aluminum garnet (QS Nd:YAG) laser and sunscreens with skin bleaching agents, particularly focusing on patient evaluation and satisfaction. This study also aims to demonstrate the influence of several factors on treatment outcomes, including gender, steroid use, sun exposure, contraceptive pills, pregnancy, remedies used, and others.

## Patients and Methods

**Fig. 1:**
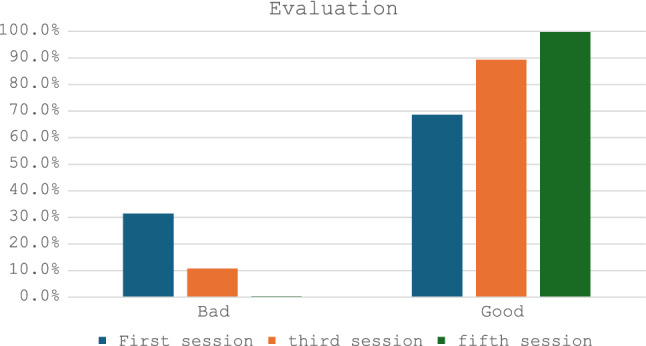
Illustration showing patient evaluation during the five sessions

A randomized cross-sectional analytical study was conducted in a private clinic to assess the effectiveness and safety of lasers for treating hyperpigmentation. The study was from early January 2022 to the end of September 2023, involving 300 patients. All patients were analyzed and assessed using several parameters (Fig. 1).

**Fig. 2:**
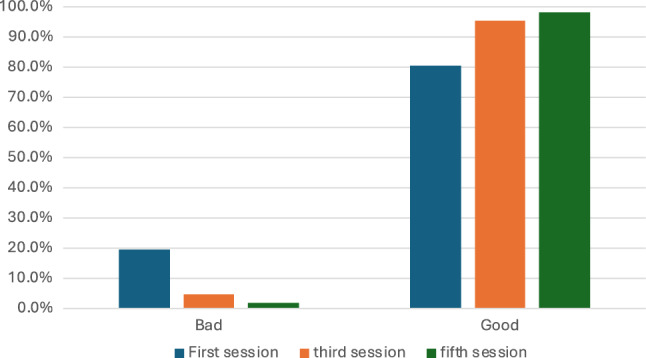
Illustration showing patient satisfaction during the five sessions

The patients were selected from a pool of cases in a database containing 965 cases according to the inclusion and exclusion criteria below (Fig. 2).

### Inclusion Criteria

1. Patients with chloasma.

2. Patients with post-acne hyperpigmentation.

3. Patients with congenital nevus.

4. Patients with post-traumatic hyperpigmentation.

5. Patients with post-burn hyperpigmentation.

6. Patients with a well-documented history, Wood's light session, and photographs.

7. Patients who underwent at least five sessions.

### Exclusion Criteria

1. Patients with missed photos during the session.

2. Patients with fewer than two laser sessions.

3. Patients with lentigo.

4. Patients with freckles.

**Fig. 3:**
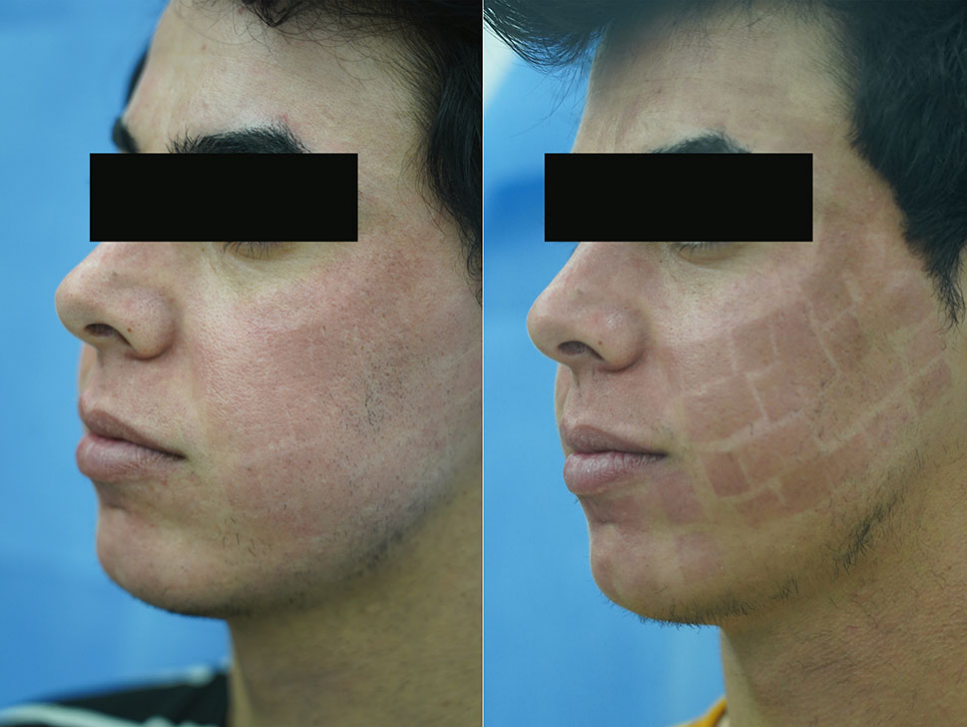
Pictures showing a patient with post-CO2 hyperpigmentation, (Right) the After effect of multimodality treatment (Left)

Outcome measurement:

Primary outcome: To evaluate the multimodality regime in the management of hyperpigmentation (Fig. 3).

Secondary outcome: To illustrate patient satisfaction with this treatment.

**Fig. 4:**
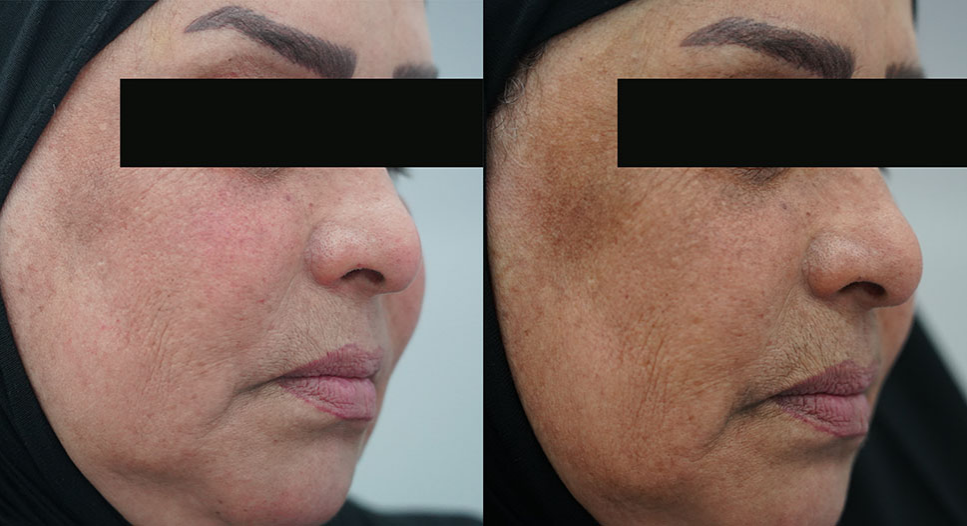
Pictures showing a patient with melasma, the effect of multimodality treatment before (Right) and (Left) after the treatment

Each patient underwent a full clinical evaluation, including a full medical assessment, regarding the patient's history for any factors such as pregnancy, sun exposure, use of specific medications, use of special remedies for measures of pigmentation, and SHOTAH (a general term in our populion representing psychological stress-induced hyperpigmentation). Wood light was used to evaluate the depth of pigmentation and the percentage of the pigments placed for each patient, in which deep pigments remained the same without any changes while superficial epidermal pigments became darker than the original under Wood's light (Fig. 4).

Clinical evaluation of outcomes was by examining pre- and post-treatment photos by three independent expert observers, rating the results as follows:

1. Worse

2. No change

3. Mild change

4. Good change

5. Excellent change

**Fig. 5:**
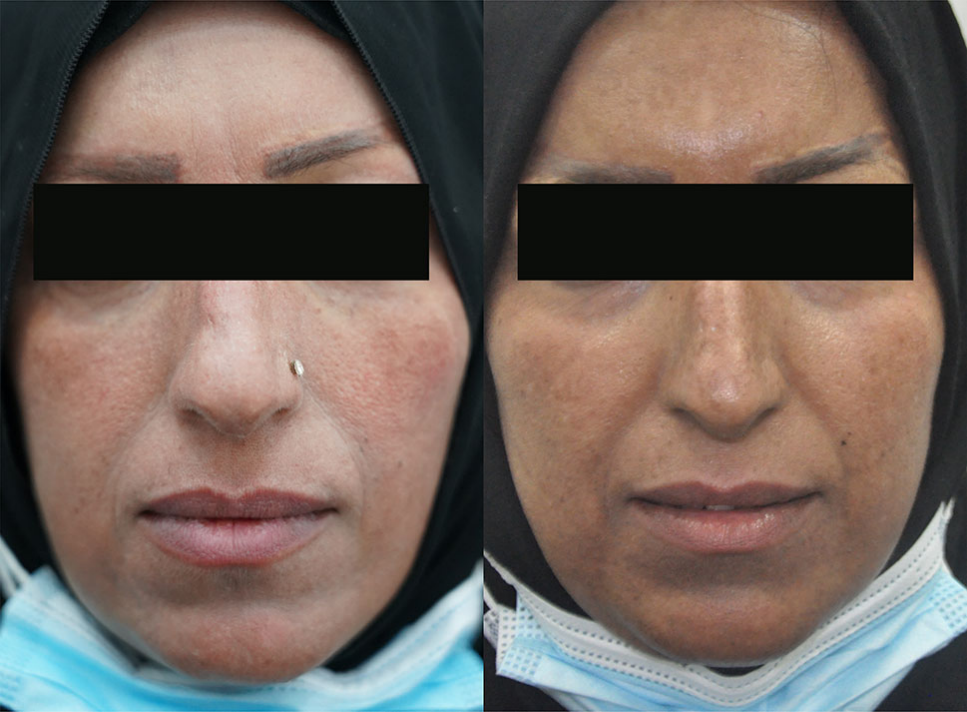
Pictures showing a patient with melasma, the effect of multimodality treatment before (Right) and (Left) after the treatment

These results were categorized into two groups for simplified statistical evaluation. So 1 & 2 will be considered “bad” results, while 3, 4, and 5 will be considered “good” results (Fig. 5).

Patient satisfaction: Measured using the Likert satisfaction scale:

1. Very dissatisfied

2. Dissatisfied

3. Neither dissatisfied nor satisfied

4. Satisfied

5. Very satisfied

These results were also simplified into two groups: “bad” includes 1, 2, & 3 while “good” includes 4 & 5.

### Laser Session

**Fig. 6:**
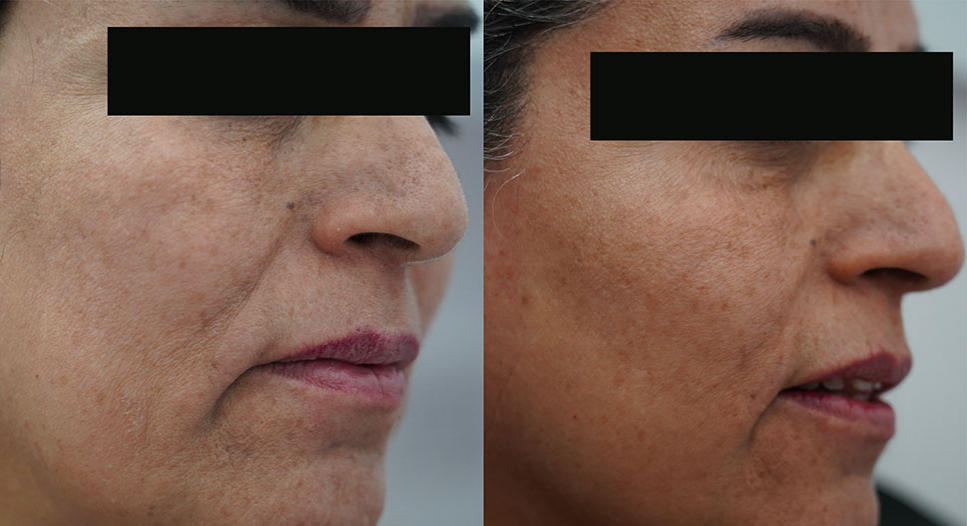
Pictures showing a patient with melasma, the effect of multimodality treatment before (Right) and (Left) after the treatment

After the patient's examination and lesion depth had been evaluated using Wood's light, a laser beam (Won-Cosjet TR ) was focused on the hyperpigmented area with a spot size (8–10 mL), wavelength of 1064 nm, fluency (0.84 J/cm2), and frequency of 8 Hz. The procedure was performed by a circular movement over the lesion until redness started to appear and induration(low-toning technique). The lesion was not washed for at least 4 hours and then kept under aloe vera overnight. Laser sessions typically consisted of a minimum of five sessions (Fig. 6).

All patients were instructed to use SPF 35 skin cream (every two hours; 8 AM, 10 AM, 12 PM, 2 PM, and 4 PM), kojic acid, and arbutin skin lightener cream (applied on the dark area at sunset for two hours), hydroquinone 4% and hydrocortisone 1% skin creams (two creams should be mixed together and applied to the darkest area at night, washed at early morning).

### Definition of Variables

**Fig. 7:**
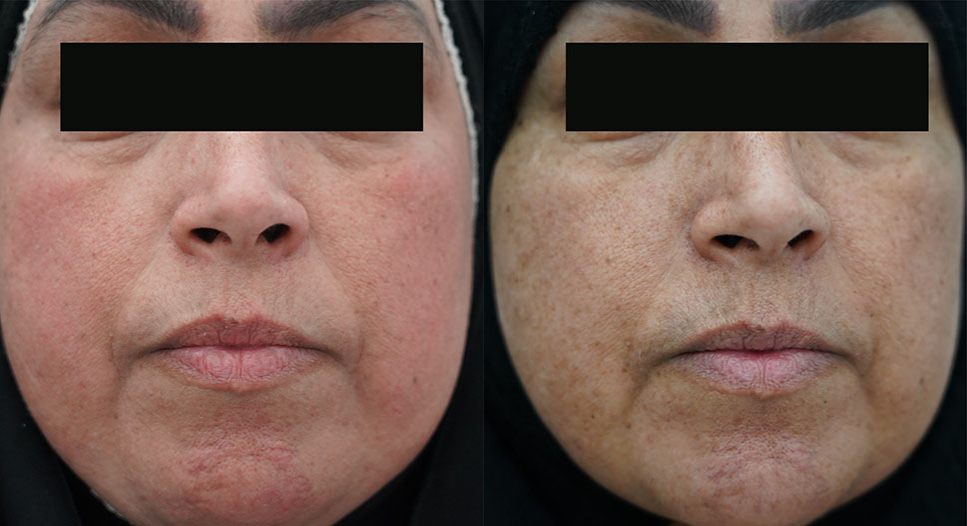
Pictures showing a patient with melasma, the effect of multimodality treatment before (A) and (B) after the treatment

SHOTAH: A state of extraordinary emotional response to a terrible event such as an accident or natural disaster (Fig. 7).

### Statistical Analysis

The data were analyzed using the Statistical Package for the Social Sciences (SPSS) version 27.0 (IBM Corp., Armonk, NY, USA). Descriptive statistics were computed for all variables to summarize the basic features of the dataset. Categorical variables were expressed as frequencies and percentages, while continuous variables were reported as means ± standard deviations or medians with interquartile ranges based on their distribution.

## Results

### General Demographic Data Distribution Among the Studied Groups

The study included 299 participants with a mean age of 36.14 ± 8.3936.14 ± 8.39 years and an average age of 36 years. The vast majority of participants were female (92.6%) compared to male (7.4%). Most individuals were complaining of melasma rather than other conditions (95.0%). The most common risk factor was sun exposure (61%), followed by SHOTAH, pregnancy, steroids, remedies and contraceptive pills (58.8%, 54%, 49.1%, and 44%, respectively; Table 1)

**Table 1:** General demographic data distribution among the studied groups

Variables		Count (No. 299)	Percentage
Age (Years)	Mean ± SD	36.14 ± 8.39	
	Minimum–Maximum	13–75	
	Median	36	
Sex	Male	22	7.4%
	Female	277	92.6%
Morbidity characteristics	Melasma	284	95.0%
	Others^*^	15	5.0%
Risk factors	Sun exposure	50	61.0%
	SHOTAH	40	58.8%
	Pregnancy	40	54.8%
	Steroid	36	54.5%
	Remedy	28	49.1%
	Contraceptive pills	33	44.0%

^*^Others: nevus of Ota, neurofibromatosis hyperpigmentation patches, post-inflammatory hyperpigmentation, post-CO2 hyperpigmentation.

Table 2 shows the distribution of responses for both evaluation and satisfaction across three different sessions. Regarding evaluation, the percentage of "Bad" evaluations decreased consistently from the first to the fifth session, with 31.4%, 10.7%, and a minimal 0.3%, respectively. Correspondingly, the percentage of "Good" evaluations increased from the first to the fifth session, starting at 68.6%, rising to 89.3%, and finally reaching 99.7%. In terms of patient satisfaction, the percentage of "Bad" ratings significantly decreased over the sessions, declining from 19.4% in the first session to 4.7% in the third session, and further to just 1.7% in the fifth session. Conversely, the percentage of "Good" patient satisfaction ratings steadily rose, starting at 80.6% in the first session, increasing to 95.3% in the third session, and peaking at 98.3% in the fifth session. These data suggest a trend of improving evaluations and increasing patient satisfaction levels as the sessions progressed, indicating an overall positive trajectory in participant feedback (Table 3).

**Table 2:** General evaluation and patients' satisfaction assessments among the three sessions

Variables		First Session	Third Session	Fifth Session
Evaluation	Bad	94 (31.4%)	32 (10.7%)	1 (0.3%)
	Good	205 (68.6%)	267 (89.3%)	298 (99.7%)
Patients' satisfaction	Bad	58 (19.4%)	14 (4.7%)	5 (1.7%)
	Good	241 (80.6%)	285 (95.3%)	294 (98.3%)

**Table 3:** Laser-related parameters among the three sessions

Variables	First Session	Third Session	Fifth Session
Wood's light (Mean ± SD)	48.71 ± 12.14	47.73 ± 11.44	42.13 ± 7.55
	40 (20–75)	40 (20–75)	40 (20 –60)
Number of shoots	7768.37 ± 2714.07	7261.37 ± 2943.63	6901.89 ± 3370.77
	9000 (300–16000)	8500 (600–14000)	8750 n(300–11000)
Duration between sessions (Mean ± SD) (Days)	------------	30.33 ± 48.32	92.69 ± 48.17

### Laser-Related Parameters Among the Three Sessions

Regarding laser-related parameters among the three sessions, the study showed that Wood's light intensity was highest in the first session and decreased in the third and fifth sessions (48.71 ± 12.14, 47.73 ± 11.44, 42.13 ± 7.55), respectively. The number of shoots was highest in the first session and decreased in the third and fifth sessions (7768.37 ± 2714.07, 7261.37 ± 2943.63, 6901.89 ± 3370.77), respectively. The duration between sessions was longest in the fifth session and shortest in the first (92.69 ± 48.17, 30.33 ± 48.32), respectively.

Table 4 provides an overview of various variables and their distribution across different sessions. In the third session, the proportion of good outcomes remained high for both males (100%) and females (99.6%). The table shows that males responded faster than females. The data indicated that male patients exhibited a notably more pronounced response to the treatment regimen compared to their female counterparts. These findings underscore the nuanced interplay of causal factors in patient outcomes and suggest potential gender-related disparities in treatment response that warrant further investigation (Table 5).

**Table 4:** General evaluation assessments of the risk factors among three sessions

Variables		First session		Third session		Fifth session	
		Bad	Good	Bad	Good	Bad	Good
Gender	Male	4 (18.2%)	18 (81.8%)	1 (4.5%)	21 (95.5%)	0 (0.0%)	22 (100.0%)
	Female	90 (32.5%)	187 (67.5%)	31 (11.2%)	246 (88.8%)	1 (0.4%)	276 (99.6%)
Steroids	Yes	1 (16.7%)	5 (83.3%)	0 (0.0%)	6 (100.0%)	0 (0.0%)	6 (100.0%)
	No	3 (60.0%)	2 (40.0%)	2 (40.0%)	3 (60.0%)	0 (0.0%)	5 (100.0%)
Sun exposure	Yes	18 (36.0%)	32 (64.0%)	7 (14.0%)	43 (86.0%)	0 (0.0%)	50 (100.0%)
	No	13 (40.6%)	19 (59.4%)	4 (12.5%)	28 (87.5%)	0 (0.0%)	32 (100.0%)
Contraceptive pills	Yes	13 (39.4%)	20 (60.6%)	4 (12.1%)	29 (87.9%)	0 (0.0%)	33 (100.0%)
	No	15 (35.7%)	27 (64.3%)	6 (14.3%)	36 (85.7%)	0 (0.0%)	42 (100.0%)
Pregnancy	Yes	13 (32.5%)	27 (67.5%)	5 (12.5%)	35 (87.5%)	0 (0.0%)	40 (100.0%)
	No	15 (45.5%)	18 (54.5%)	5 (15.2%)	28 (84.8%)	0 (0.0%)	33 (100.0%)
SHOTAH	Yes	14 (35.0%)	26 (65.0%)	7 (17.5%)	33 (82.5%)	0 (0.0%)	40 (100.0%)
	No	12 (42.9%)	16 (57.1%)	4 (14.3%)	24 (85.7%)	0 (0.0%)	28 (100.0%)
Remedy	Yes	7 (25.0%)	21 (75.0%)	3 (10.7%)	25 (89.3%)	0 (0.0%)	28 (100.0%)
	No	11 (37.9%)	18 (62.1%)	3 (10.3%)	26 (89.7%)	0 (0.0%)	29 (100.0%)

**Table 5:** General patient satisfaction assessments of the risk factors among three sessions

Variables		First session		Third session		Fifth session	
		Bad	Good	Bad	Good	Bad	Good
Gender	Male	2 (9.1%)	20 (90.9%)	0 (0.0%)	22 (100.0%)	1 (4.5%)	21 (95.5%)
	Female	56 (20.2%)	221 (79.8%)	14 (5.1%)	263 (94.9%)	4 (1.4%)	273 (98.6%)
Steroids	Yes	1 (16.7%)	5 (83.3%)	0 (0.0%)	6 (100.0%)		
	No	1 (20.0%)	4 (80.0%)	0 (0.0%)	5 (100.0%)		
Sun exposure	Yes	5 (10.0%)	45 (90.0%)	2 (4.0%)	48 (96.0%)	1 (2.0%)	49 (98.0%)
	No	4 (12.5%)	28 (87.5%)	0 (0.0%)	32 (100.0%)	0 (0.0%)	32 (100.0%)
Contraceptive pills	Yes	4 (12.1%)	29 (87.9%)	1 (3.0%)	32 (97.0%)	1 (3.0%)	32 (97.0%)
	No	3 (7.1%)	39 (92.9%)	1 (2.4%)	41 (97.6%)	0 (0.0%)	42 (100.0%)
Pregnancy	Yes	4 (10.0%)	36 (90.0%)	1 (2.5%)	39 (97.5%)	1 (2.5%)	39 (97.5%)
	No	4 (12.1%)	29 (87.9%)	1 (3.0%)	32 (97.0%)	0 (0.0%)	33 (100.0%)
SHOTAH	Yes	4 (10.0%)	36 (90.0%)	1 (2.5%)	39 (97.5%)	0 (0.0%)	40 (100.0%)
	No	3 (10.7%)	25 (89.3%)	1 (3.6%)	27 (96.4%)	1 (3.6%)	27 (96.4%)
Remedy	Yes	1 (3.6%)	27 (96.4%)	0 (0.0%)	28 (100.0%)	0 (0.0%)	28 (100.0%)
	No	2 (6.9%)	27 (93.1%)	0 (0.0%)	29 (100.0%)	0 (0.0%)	29 (100.0%)

The steroid risk factor showed that steroid users had a higher and faster response rate than non-steroid users to the multimodality regime.

Comparable results were noted for sun exposure, contraceptive pills, and pregnancy in both exposed and non-exposed groups throughout the sessions.

For the variable "SHOTAH," most individuals with this characteristic had good outcomes in the first session (65.0%), while non-SHOTA had only a 57.1% response in the first session, indicating a faster response.

Patients using mask remedies showed a faster and higher response in the first sessions than non-remedy users did, 75% versus 62.1%.

Broadly, the analysis did not reveal any obvious impacts of various causative agents on patients' evaluation, assessment, and response.

In general, logistic regression statistical analysis did not find any clear effects of different factors on patient satisfaction.

## Discussion

Skin hyperpigmentation is a common dermatological concern that affects individuals of diverse backgrounds and demographics. The search for effective treatment modalities has led to the exploration of various laser therapies, including the Nd:YAG (neodymium-doped yttrium aluminum garnet) laser. In this study, we investigated the efficacy of the Nd:YAG laser in managing skin hyperpigmentation, focusing on the influence of gender, steroid usage, sun exposure, contraceptive pill usage, pregnancy status, history of SHOTAH, and remedy usage across multiple treatment sessions. Numerous contributing factors have been associated with the development of melasma, including ultraviolet (UV) exposure, hormonal influences such as oral contraceptives and pregnancy, drugs, and cosmetics. In this study, 61% of patients reported a history of significant sun exposure, a figure similar to the study by Al-Hamdi K et al., in which 59.69% of the population had significant sun exposure. UV exposure is a recognized major factor in melasma development, as sunlight can trigger lipid peroxidation in cell membranes, generating free radicals that stimulate melanogenesis, which was notably higher among pregnant women (54.8%) in our study, compared to 37.9% in Al-Hamdi K et al.'s study. Other significant factors influencing melasma prevalence included drugs and cosmetics (49.1%) and emotional triggers (58.8%) compared to 18.4% and 40.3%, respectively in Al-Hamdi K et al.'s study [16].

Contraceptive pill usage was noted in 44% of our patients, surpassing the prevalence reported in previous research, which represents 23.96%. Hormonal changes during pregnancy, such as increased estrogen, progesterone, and melanocyte-stimulating hormone levels, likely contribute to these findings [16].

Regarding evaluation scores in this study, some participants initially recorded a "no response" assessment (10.7%, 0.3%), but the majority achieved a "good response" (89.3%, 99.7%) after the third and fifth sessions. The highest patient satisfaction scores were recorded in the fifth session, followed by the third, while some participants assessed their satisfaction as "no response" after the first session, achieving 95% and 98% respectively in the third and fifth sessions. The use of low-toning lasers is much safer with optimum results and almost no complications compared to the high-toning lasers.

In comparison with our findings, Cho et al., utilizing laser only, reported that 70% of patients experienced marked improvement, with 28% achieving near-total improvement, and only 5% reported worsening of the pigmentation [27].

Wattana Krai et al. observed significant improvements (92.5%) in the relative lightness index with combined treatment using Q-switched Nd:YAG and hydroquinone [28].

Lee et al. treated 50 patients with Q-switched Nd:YAG and reported 50%–74% improvement with no serious side effects [29].

However, concerns regarding mottled hypopigmentation with low-fluence QSNY treatments have been raised, with some authors reporting a high incidence of this adverse event, while no such complications were reported in this study [30].

Menaker et al. treated 10 patients with a novel 1320-nm Nd:YAG laser in a series of three treatments at two-week intervals. Three months after treatment, 4 of 10 patients improved, but the results were not statistically significant, and three patients developed pitted scarring as a complication from the laser treatments, an adverse effect not reported in this study [31].

In a study by Madhulika A. Mhatre, the QS Nd:YAG was assessed for treating facial pigmentary lesions in Indian patients. While recognized for treating tattoos and various pigmentary issues, its efficacy for facial lesions remains less explored. The study involved 100 patients, primarily with PIH. Regarding melasma, 69% showed varying levels of improvement, 26% had no change, and 5% experienced worsening pigmentation. This highlights the QS Nd:YAG laser's varied outcomes and the need for clear patient counseling and potential refinements in treatment approaches [32].

The inclusion of a patient's history of SHOTAH as a potential risk factor for hyperpigmentation is a crucial aspect of this study that deserves special attention. Stressful events have been identified as initiating factors in 4–7% of cases and worsening factors in around 26% of cases in Brazil [33].

As our findings demonstrated, individuals with a history of SHOTAH (40 [58.8%]) showed increasing patient satisfaction with each evaluation, rising to almost all patients during the third to fifth session evaluation.

This observation highlights the significance of considering SHOTAH history when evaluating and tailoring treatment approaches for patients with hyperpigmentation concerns.

In this study, gender-based differences in initial treatment responses were evident. Initially, male patients displayed a faster response to Nd:YAG laser treatment than females did. However, as treatment sessions advanced, these gender disparities gradually diminished, resulting in comparable rates of success and patient satisfaction.

Steroid use also influenced initial responses, with steroid users experiencing a faster start in treatment. Over time, the gap between steroid users and non-users closed, indicating that steroid use did not clinically impact overall patient evaluation and satisfaction. While initial treatment outcomes varied based on sun exposure history, these differences waned as therapy progressed. Patients using contraceptive pills or those pregnant exhibited slightly lower initial responses, which lessened as treatment sessions continued.

In summary, the initially faster response seen in male patients and steroid users, along with the various factors we examined did not significantly impact patient evaluation and satisfaction levels. These results emphasize the importance of personalized care in dermatology and plastic surgery, in addition to the utilization of multimodality treatment including sunscreen, hydroquinone, and laser therapy. Clinicians should consider individual patient characteristics and tailor treatment approaches accordingly. Further research is warranted to delve into the underlying mechanisms driving these observations, allowing us to refine treatment protocols and enhance patient satisfaction in the management of skin hyperpigmentation.

### Limitiations of the Study

The use of Wood's light as an evaluation tool for assessing the severity of the condition is a subjective method that depends on the operator's expertise. However, with advancements in technology, more objective and standardized tools are now available to evaluate the extent and depth of pigmentation. These modern technologies minimize operator dependency and reduce bias, leading to more accurate assessments. In contemporary plastic surgery, technological innovations are playing an increasing role not only in the preliminary evaluation of various conditions but also in predicting prognosis, ultimately improving patient outcomes.

Patient-reported outcome measures (PROMs) play a crucial role in modern plastic surgery by providing standardized assessments of patient satisfaction and quality of life across various procedures. Unlike general satisfaction scales such as the Likert scale, validated PROMs—such as BREAST-Q [34] for breast surgery, SCAR-Q [35, 36] for scar assessment, and FACE-Q [37] for facial aesthetic procedures—offer more comprehensive and reliable insights into patient experiences. These tools are widely used in both aesthetic and reconstructive surgery to evaluate outcomes, guide clinical decision-making, and improve patient counseling. While our study utilized the Likert satisfaction scale for simplicity, future research could benefit from incorporating validated PROMs to ensure a more detailed and comparable assessment of patient-reported outcomes.

## Conclusion

Proper and thorough evaluation of hyperpigmentation and its causes dictates the treatment approach. Multimodality treatment of hyperpigmentation results in superior outcomes compared to a single modality of treatment. Evaluation and satisfaction across different laser sessions show the percentage of bad evaluations from the first towards the fifth session.

### Recommendation

The use of multimodality management for hyperpigmentation has improved results and patient satisfaction.
